# Perceived Vulnerability to Disease Questionnaire: psychometric validation with a Portuguese sample

**DOI:** 10.1186/s40359-022-00838-0

**Published:** 2022-05-22

**Authors:** Jacqueline Ferreira, Ana C. Magalhães, Pedro Bem-Haja, Laura Alho, Carlos F. Silva, Sandra C. Soares

**Affiliations:** 1 CINTESIS@RISE, Department of Education and Psychology, University of Aveiro, Aveiro, Portugal; 2 William James Center for Research, University of Aveiro, Aveiro, Portugal; 3 Department of Education and Psychology, University of Aveiro, Campus Universitário de Santiago, 3810-193 Aveiro, Portugal; 4 Mind - Instituto de Psicologia Clínica e Forense, Lisbon, Portugal

**Keywords:** Perceived vulnerability to disease, Disease avoidance, Perceived infectability, Germ aversion, Individual differences

## Abstract

**Background:**

Individual differences in one’s perceived vulnerability to infectious diseases are implicated in psychological distress, social and behavioral disease avoidance phenomena. The Perceived Vulnerability to Disease Questionnaire (PVD) is the most extensively used measure when it comes to assessing subjective vulnerability to infectious diseases. However, this measure is not yet accessible to the Portuguese population. The present study aimed to adapt and validate the PVD with 136 Portuguese participants.

**Methods:**

Factorial, convergent and discriminant validity (of both the scale and between each factor), and reliability analysis were assessed.

**Results:**

A modified bifactorial model, comprised of Perceived Infectability and Germ Aversion factors, was obtained, with acceptable goodness-of-fit indices, adequate convergent and discriminant validity, and good internal consistencies.

**Conclusions:**

Overall, the 10-items European-Portuguese PVD appears to be a reliable and valid measure of one’s perceived vulnerability to disease, with potential relevance for application in both research and clinical practice pertaining to disease-avoidance processes.

**Supplementary Information:**

The online version contains supplementary material available at 10.1186/s40359-022-00838-0.

## Background

Humans, like other animals, possess specialized systems that protect them from pathogens, which pose high risks for the survival and reproductive fitness of the species [1, 2]. One of these systems is the biological immune system, responsible for detecting and destroying foreign elements once they enter the body. However, this complex set of responses is merely reactive, occurring only after an infection has settled, and entails some constraints for the individual, namely the high consumption of physiological energy and the loss of social opportunities (e.g., forming friendship/romantic relationships) [3, 4]. Thus, having a system capable of avoiding pathogens before they enter the body would reduce such costs.

The behavioral immune system (BIS) provides an early defensive response against pathogens. It is extremely sensitive to any cues (be it in objects or people) that suggest the presence of disease in the environment, even if they do not represent a real risk of contagion [5, 6]. Once detected, these cues trigger a disgust reaction, cognitive biases and behavioral avoidance responses with the ultimate goal of inhibiting contact with pathogens [4].

The intensity of these responses is influenced not only by the salience of disease cues, but also by individual differences. For instance, Mortensen et al. [7] showed that a high disease salience (disease versus neutral prime) can temporarily influence individual’s personality traits by making them perceive themselves as less extroverted and open to experience. These traits reflect less willingness to engage in social interactions, a response closely linked to the BIS. Moreover, these effects were more prominent in individuals who perceived themselves as more vulnerable to disease. This is in line with one assumption of the BIS, which states that individuals who are—or merely perceive themselves to be—more vulnerable to infection, display stronger aversive responses towards pathogen-connoting stimuli [5].

Different types of self-report instruments used to assess perceived vulnerability to disease exist, but only one reliably measures individual differences in perceived vulnerability to infectious diseases. For instance, measures of disgust sensitivity can be used to that end, but these tend to focus on a broad range of stimuli of which only a subset are directly relevant to disease transmission. Likewise, instruments designed to assess hypochondria and other health-relevant anxieties involve several potential health problems, not only infectious diseases [8]. Thus, while these measures can be used to infer perceived vulnerability to disease, they are not specific to infectious diseases, unlike the Perceived Vulnerability to Disease Questionnaire (PVD).

The PVD is widely used and was first introduced by Park et al. [9]. Throughout the years, it underwent multiple revisions (14 to 19 items) until Duncan and collaborators [8] developed and validated a 15-item version of the scale, which assesses one’s beliefs about personal susceptibility to and emotional discomfort associated with a potential contagion from infectious diseases. This version has shown good psychometric properties and can be used with the general population.

Studies using the PVD have been consistently showing that individuals chronically concerned about disease transmission seem to be more sensitive to cues heuristically associated with disease and, consequently, tend to adopt more overt discriminant behaviors towards others perceived as having a poor health status, like the obese, the elderly or people with physical disabilities [9–11]. They also tend to show more ethnocentric and xenophobic attitudes against strangers [12, 13]. Thus, despite the protective role of the BIS, it also seems to contribute to aversive responses towards people associated with a risk for contagion [2, 3]. Furthermore, while perceived vulnerability to disease leads people to engage more fully in proactive preventative behaviors, especially beneficial when the risk of contagion is high (e.g., Covid-19 pandemic), it has also been associated with considerable psychological distress, increasing both anxiety and depression levels [14, 15]. However, no instrument to measure the perceived vulnerability to infectious diseases exists for the Portuguese population.

Understanding how this variable contributes to the inhibition of social interactions can be helpful for the development of better social strategies aimed at dealing not only with negative behaviors (e.g., stigmatization or prejudice) against people who are (or appear to be) ill, but also with the increased psychological distress felt by those who regard themselves as more susceptible to diseases. Hence, this instrument has potential relevance for application in both research and clinical practice pertaining to disease-avoidance processes. With this in mind, the main goal of this study was to adapt and validate the PVD for the Portuguese population.

## Methods

### Participants

One-hundred ninety-five participants, aged between 18 and 65 years (155 women, M = 26.16, SD = 8.86) from three Portuguese Universities voluntarily filled the online protocol. Fifty-nine were excluded because they did not fully complete it. The final sample included 136 participants (109 women, M = 27.01, SD = 9.77).

### Instruments

The Perceived Vulnerability to Disease Questionnaire (PVD) [8], a 15-item seven-points scale (one = “Strongly Disagree” to seven = “Strongly Agree”) with two factors, “Perceived Infectability” (PI) and “Germ Aversion” (GA), was completed.

Participants also completed the Disgust Propensity and Sensitivity Scale-Revised (DPSS-R) [16], a 11-item five-points scale divided into two subscales: “Disgust Propensity” and “Disgust Sensitivity”; the Disgust Scale-Revised (DS-R) [17], a 27-item five-points scale measuring disgust in three dimensions: “Core Disgust”, “Animal-reminder Disgust” and “Contamination-based Disgust”; the Maudsley Obsessive Compulsive Inventory (MOCI) [18], with 30 true/false items measuring obsessive–compulsive symptoms in three subscales: “Doubting and Rumination”, “Checking” and “Cleaning”; the Spider Phobia Questionnaire-Revised (SPQ-R15) [19], with 15 true/false items measuring fear and avoidance of spiders; the Minnesota Multiphasic Personality Inventory–2 Hypochondria subscale (MMPI-2 Hs) [20], with true/false items assessing hypochondria symptoms and physical well-being; and the NEO-Five Factor Inventory (NEO-FFI) [21], a 60 yes/no measure assessing Neuroticism, Extraversion, Openness, Agreeableness and Conscientiousness.

### Procedure

The PVD was adapted to Portuguese using the translation/back-translation methodology [22]. First, it was translated into European-Portuguese by two bilingual individuals and reviewed by a highly proficient in English researcher. Afterwards, it was submitted to a think-aloud procedure, back-translated by a bilingual researcher and sent to the original authors for final approval of the Portuguese version.

The entire protocol was available online. The access link was sent to three Universities and divulged through their staff/student mailing lists. All participants were asked to read the instructions, provide informed consent and fill out the scales and sociodemographic information.

### Statistical Analysis

All analysis were performed using R [23]. The following packages were used: highr [24], rio [25], psych [26], GPArotation [27], EFAtools [28], readxl [29] and lavaan [30]. See Additional file 1 for a more detailed analysis outline and additional results.

Mardia's Test was performed to assess multivariate normality of the sample [31] and the Jöreskog and Sörbom [32, p. 171] equation (see below) to verify the adequacy of the sample size for the factor analysis.

$$Number\,of\,Participants = \frac{{\left( {p + 1} \right)\left( {p + 2} \right)}}{2}$$

where *p* is the number of observed variables.

Construct validity was evaluated by calculating its three sub-components: Factorial, convergent and discriminant validity. Since versions from different countries show inconsistent factorial structures, an Exploratory Factor Analysis (EFA) followed by a Confirmatory Factor Analysis (CFA) using weighted least-square-mean and variance adjusted estimator (WLSMV) were conducted. Several CFAs were compared to verify the factor structure that best fits the data. The overall goodness-of-fit was assessed using the following indexes and cut-off points for “good adjustment”: Chi-square (χ^2^); Comparative Fit Index (CFI; 0.90 ≤ CFI ≤ 0.95); Tucker-Lewis Index (TLI; 0.90 ≤ TLI ≤ 0.95); Root Mean Square Error of Approximation (RMSEA; 0.05 ≤ RMSEA ≤ 0.70); P[rmsea ≤ 0.05]; and Standardized Root-Mean-Residual (SRMR; SRMR < 0.80) [33].

Convergent and discriminant validity between the two PVD factors, and of the scale, were assessed using the Fornell and Larcker [34] method and the correlational method, respectively. Thus, to show convergent validity of the scale, positive and significant Spearman correlations between PVD factors and both DPSS-R subscales, DS-R (total score, Core Disgust and Contamination-based Disgust subscales), MOCI, MMPI-2 Hs and NEO-FFI Neuroticism were expected. Particularly, a stronger correlation between GA and disgust propensity (i.e., DPSS-R Disgust Propensity, and DS-R subscales, especially Contamination-based Disgust), GA and MOCI, and PI and MMPI-Hs were expected. Conversely, discriminant validity of the scale was expected to result in low or non-significant correlations between PVD factors and DS-R Animal-reminder Disgust subscale, SPQ-R15 and NEO-FFI subscales (except Neuroticism).

Considering the ordinal nature of the data, a reliability analysis using Ordinal Cronbach's alpha, based on polychoric correlation, was performed to test the internal consistency of the factors.

## Results

Mardia’s test showed that data is not multivariate normal, g1p = 34.23, χSkew = 1243.78, *p* < 0.001; g2p = 281.09, ZKurtosis = 8.53, *p* < 0.001; χSMSkew = 1263.08, *p* < 0.001. Sample size was considered adequate for factorial analysis.

### Factorial validity

Bartlett's test of sphericity was significant, χ^2^(105) = 599.93, *p* < 0.001, and the Kaiser–Meyer–Olkin Measure of Sampling Adequacy (KMO) analysis returned a value of 0.81 for the overall matrix, and values between 0.65 and 0.96 for all variables. Both indicators support factor analysis as a useful approach to the data.

**Fig. 1:**
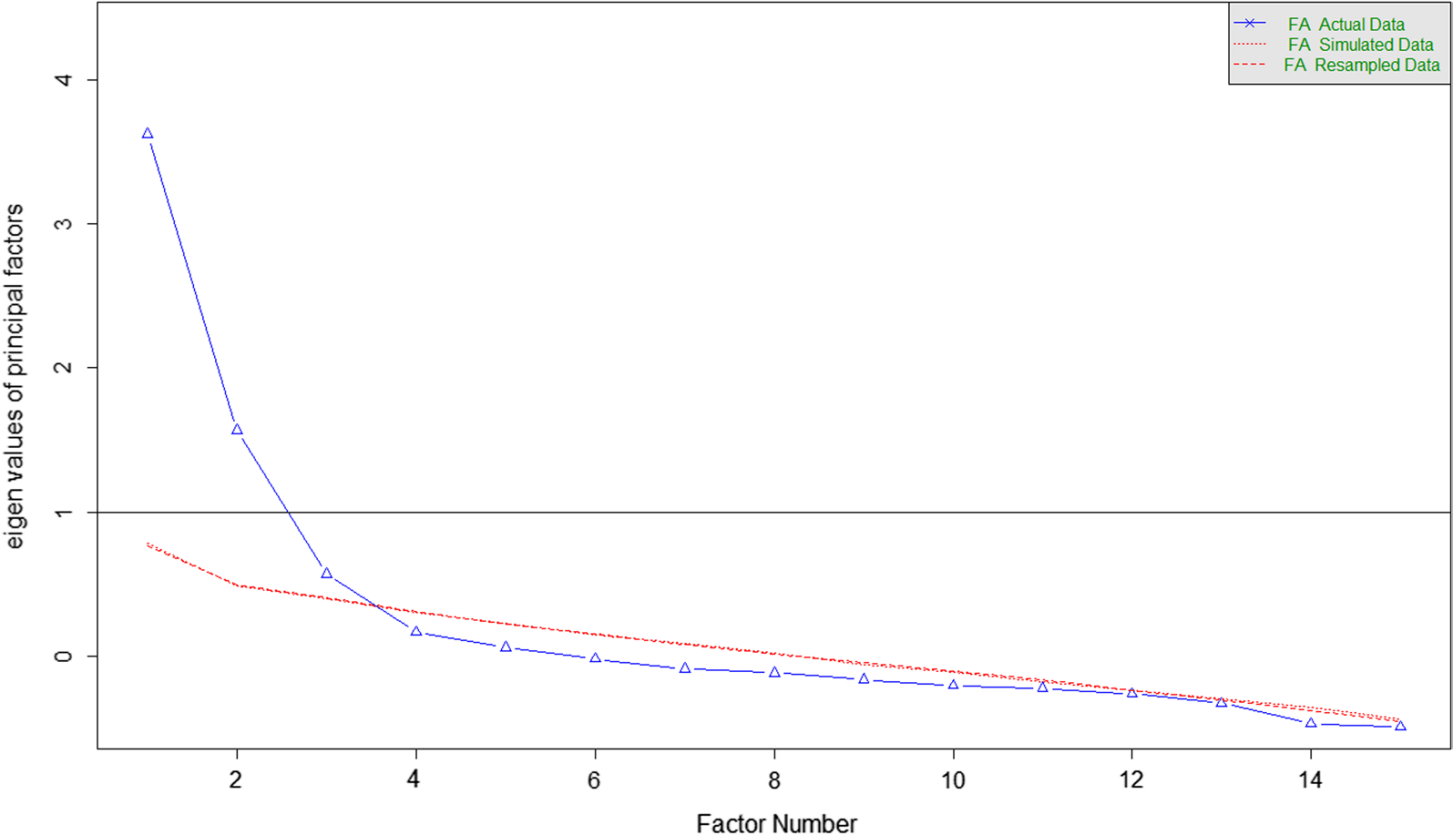
Parallel analysis scree plot with ULS estimator

Parallel analysis with unweighted least-squares estimator (ULS) indicated that three factors should be retained (see Fig. 1). However, only two principal factor eigenvalues reached values greater than 1 or 0.7 (old and new Keiser Criterion, respectively). Moreover, Hull method with CFI and RMSEA, and lower bound of RMSEA 90% CI, also support a two-factor retention. Given these results and the conceptual framework surrounding the bifactorial structure of the original scale, a two-factor solution was extracted.

An EFA with Promax rotation using ULS estimator was performed. Factor loadings and respective R^2^, Uniqueness and Complexity by factor are shown in Table 1.

**Table 1:** Factor loadings and respective R^2^, uniqueness and complexity values

	Factor		R^2^	Uniqueness	Complexity	CL Ratio
	F1(PI)	F2(GA)				
Q1	− 0.09	**0.68**	0.43	0.57	1.0	7.6
**Q2**	**0.43**^**LL**^	0.31	0.38	0.62	**1.8**	**1.4**^**CL**^
Q3	− 0.15	**0.57**	0.29	0.71	1.1	3.8
Q4	0.01	**0.60**	0.37	0.63	1.0	60
**Q5**	**0.55**	− 0.30	0.28	0.72	**1.6**	**1.8**^**CL**^
**Q6**	**0.44**^**LL**^	0.04	0.21	0.79	1.0	11
Q7	0.01	**0.68**	0.47	0.53	1.0	68
Q8	**0.84**	− 0.13	0.64	0.36	1.1	6.5
**Q9**	0.01	**0.41**^**LL**^	0.17	0.83	1.0	41
Q10	**0.58**	0.22	0.47	0.53	1.3	2.6
Q11	0.00	**0.57**	0.33	0.67	1.0	57
Q12	**0.70**	0.01	0.49	0.51	1.0	70
**Q13**	0.19	**0.38**^**LL**^	0.23	0.77	**1.5**	**2**^**CL**^
Q14	**0.59**	0.07	0.38	0.62	1.0	8.4
Q15	0.05	**0.59**	0.37	0.63	1.0	11.8

Items corresponding to each factor are listed according to the strength of their factor loading

Items deemed problematic are underlined

The results marked in bold correspond to the highest factor loading value per item, complexity and CL ratio values above the recommended, evidencing cross-loading

PI, perceived infectability; GA, germ aversion; CL ratio, primary/secondary loading; LL, loading below 0.5; CL, cross-loading

The two-factor solution accounted for 37% of the variance, with the PI factor explaining 18% and the GA factor 19% of the variance. The inter-factor correlation was 0.35. Interestingly, this factorial solution mimics the two-factor solution expected and postulated in the literature (e.g., [8]), with the highest loading of each item saturated in the theoretically correct factor.

**Fig. 2:**
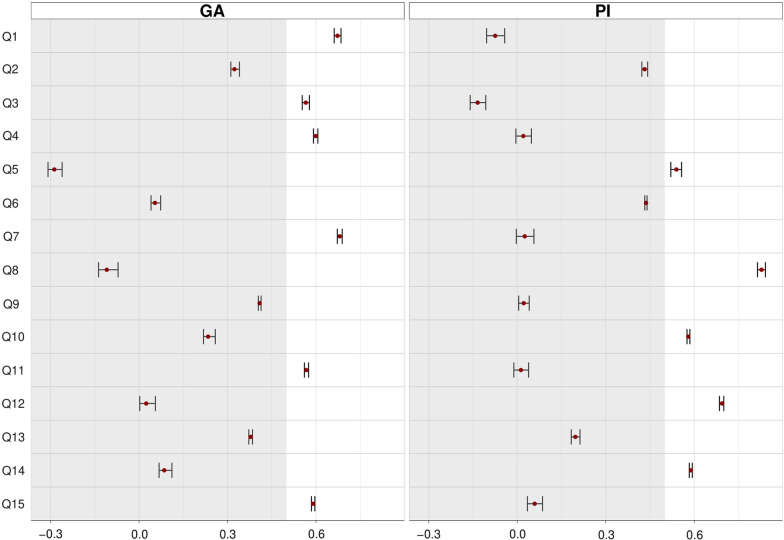
Average, maximum, and minimum loadings for each item per factor. *Note*: GA = Germ Aversion; PI = Perceived Infectability

Items Q2, Q6, Q9 and Q13 registered low loadings for the sample size (< 0.50) and some also showed cross-loadings with complexity and/or CL ratio values above those recommended [35, 36]. Despite an acceptable primary loading, item Q5 showed inadequate complexity and CL ratio, evidencing cross-loading. An average EFA with different oblique rotation models was performed to ensure elimination decision. Visual analysis of loadings distribution suggested an absence of major fluctuations (see Fig. 2). Although visually adequate, the ratio between loadings in item Q5 is still below the recommended (< 0.03) [36].

Furthermore, two Polytomous Item Response Theory analysis using generalized partial credit model—one for each factor—were performed (see Table 2). Results showed that the five aforementioned items reached discrimination values below the acceptable (≥ 0.70) [37], suggesting that they are not good at discriminating the latent trait and, therefore, supporting their removal.

**Table 2:** Results of the PIRT analysis using Generalized Partial Credit Model per factor

Item	a	b1	b2	b3	b4	b5	b6
**PI**							
Q8	1.923	− 1.291	0.412	0.555	0.648	1.405	2.168
Q10	1.113	− 0.902	1.497	0.326	2.984	0.719	2.37
Q12	1.007	− 3.341	− 0.347	0.101	0.368	1.232	2.045
Q14	0.7	− 2.772	− 0.5	− 0.219	1.044	2.667	1.377
Q2	0.562	− 1.256	1.656	0.281	1.997	3.852	1.318
Q6	0.425	− 0.963	3.325	− 0.416	2	6.297	− 1.44
Q5	0.31	− 3.701	− 0.189	− 0.637	2.211	0.023	2.199
**GA**							
Q1	1.234	− 1.891	− 1.952	− 1.419	− 1.734	− 0.749	0.476
Q7	1.093	− 0.284	1.034	0.75	1.149	2.399	3.239
Q15	0.513	0.298	0.961	− 0.659	2.148	3.051	0.839
Q4	0.465	− 1.704	0.273	0.247	− 0.689	0.237	1.611
Q11	0.449	− 1.279	0.417	1.284	− 2	2.384	0.837
Q3	0.422	− 2.268	− 0.802	0.348	1.044	0.262	0.067
Q13	0.386	− 3.475	0.881	1.476	0.234	1.961	4.371
Q9	0.234	− 1.348	0.674	0.614	0.395	0.104	1.327

Items deemed problematic are underlined

Bold to highlight that they are the factors of the items appearing below

a, discrimination ability; PI, perceived infectability; GA, germ aversion

**Fig. 3:**
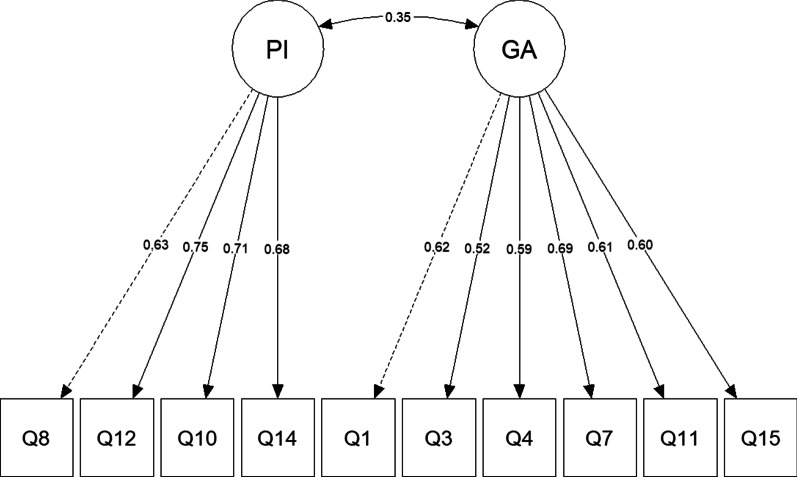
Diagram of two-factor structure (10 items) obtained using CFA with WLSMV estimator. *Note*: PI = Perceived Infectability; GA = Germ Aversion

A CFA with WLSMV was used to confirm the 10-items bifactorial structure obtained from the EFA. Results revealed an acceptable global adjustment, χ^2^(34) = 46.68; CFI = 0.93; TLI = 0.91; RMSEA = 0.05, RMSEA 90% CI [0.00, 0.09]; SRMR = 0.07. Moreover, all items reached high factor weights and appropriate individual reliabilities on latent variables (see Fig. 3).

Several CFAs were also compared to verify the factor structure that best fits the data. Apart from the original factor structure, and considering the cultural proximity, structure models from two Spanish studies assessing the psychometric properties of the PVD were tested with our sample. All models are present in Table 3.

**Table 3:** Confirmatory Factor Analysis for all the models tested

	Model 1	Model 2.1 Original	Model 2.2 Spain1	Model 2.3 Portugal	Model 3.1 Spain2	Model 3.2 Portugal	Model 4.1 Spain2	Model 4.2 Portugal
	One Factor PVD (all items)	Two Factors PI & GA (all items)	Two Factors PI & GA (w/o reverse items)	Two Factors PI & GA (w/o items Q2, Q5, Q6, Q9 and Q13)	One Factor PI (all items)	One Factor PI (w/o items Q2, Q5 and Q6)	One Factor GA (all items)	One Factor GA (w/o items Q9 and Q13)
χ^2^; *p*(df)	237.68; *p* < .001(90)	147.43; *p* < .001(89)	34.57; *p* = .12(26)	46.68; *p* = .07(34)	35.97; *p* = .001(14)	11.57; *p* < .01(2)	17.51; *p* = .62(20)	5.375; *p* = .80(9)
CFI	.50	.80	.95	.93	.84	.88	1.00	1.00
TLI	.42	.77	.93	.91	.76	.63	1.02	1.04
RMSEA (90%CI)	.11 (.09, .13)	.07 (.05, .09)	.05 (0, .09)	.05 (0, .09)	.11 (.07, .15)	.19 (.09, .30)	0 (0, .06)	0 (0, .06)
SRMR	.12	.09	.06	.07	.08	.07	.04	.03
Loadings range	(.13 to .62)	PI (.28 to .75) GA (.42 to .70)	PI (.47 to .83) GA (.41 to .69)	PI (.63 to .75) GA (.52 to .70)	(.41 to .78)	(.61 to .79)	(.40 to .69)	(.55 to .67)
Items below 0.5	Q3, Q5, Q6, Q8, Q9, Q12, Q13, Q14	PI – Q5, Q6 GA – Q3, Q9	PI – Q6 GA – Q9		Q5		Q9, Q13	

WLSMV was used as an estimator for all models

PI, perceived infectability; GA, germ aversion; Spain1, study by Magallares et al. [39]; Spain2, study by Díaz et al. [40]

Models 1, 2.1 and 3.1 obtained inadequate global and local adjustment values. Models 2.2 and 4.1, on the other hand, obtained acceptable values of global adjustment, but inappropriate local adjustment values. The remaining three models (i.e., 2.3, 3.2 and 4.2) reached acceptable values of global and local adjustments. Considering the conceptual framework and the original structure of the PVD, the bifactorial model obtained from the EFA (i.e., model 2.3) was adopted (see Additional file 2 for the original and final Portuguese version of the scale).

### Convergent and discriminant validity of PVD factors

The Average Variance Extracted (AVE) and Composite Reliability (CR) values for both factors were as follows: AVE_PI_ = 0.41, AVE_GA_ = 0.33, CR_PI_ > 0.79, CR_GA_ > 0.74. While these AVE values are below those that are usually regarded as adequate [38], Fornell and Larcker [34] state that if the AVE values are less than 0.5, but the CR values are higher than 0.6, convergent validity of the construct is still considered adequate. Furthermore, both CR values are greater than 0.7, supporting the notion of an appropriate construct reliability. Thus, convergent validity of PVD factors was confirmed.

Furthermore, both AVE values were above the square of the correlation between the two factors (0.12), indicating only 12.3% of common information between them and confirming the discriminant validity of the two-factor model [34].

### Convergent and discriminant validity of the scale

Regarding convergent validity, as shown in Table 4, both PVD subscales significantly correlated with DPSS-R subscales, MOCI, MMPI-Hs and NEO-FFI Neuroticism, while DS-R total score, Core Disgust and Contamination-based Disgust subscales only correlated with GA. Furthermore, DPSS-R Disgust Propensity subscale and MOCI correlated more strongly with GA, and MMPI-Hs with PI, as predicted.

**Table 4:** Polychoric Correlation Matrix among study variables

Variables	1	2	3	4	5	6	7	8	9	10	11	12	13	14	15	16
PVD																
1. Perceived infectability	(.82)															
2. Germ aversion	.27***	(.82)														
DPSS-R																
3. Disgust propensity	.25**	.34***	(.85)													
4. Disgust sensitivity	.29***	.22*	.50***	(.87)												
DS-R																
5. Core disgust	.04	.28***	.31***	.40***	(.81)											
6. Animal-reminder disgust	.02	.11	.20*	.39***	.68***	(.82)										
7. Contamination-based	.12	.41***	.16	.25**	.54***	.37***	(.58^a^)									
8. Total	.00	.28***	.28***	.43***	.93***	.86***	.66***	(.89)								
MOCI																
9. Total	.39***	.42***	.43***	.35***	.18^*^	.12	.25**	.20*	(.86)							
SQ-R15																
10. Total	.12	.09	.38***	.27**	.30***	.18*	.15	.27**	.37***	(.89)						
MMPI-Hs																
11. Total	.32***	.23**	.41***	.27**	.13	.14	.10	.15	.52***	.29***	(.94)					
NEO-FFI																
12. Neuroticism	.26**	.17*	.32***	.35***	.20*	.12	.11	.18*	.54***	.37***	.57***	(.88)				
13. Extraversion	.15	.11	.10	.01	.06	.11	− .07	.06	.24**	− .08	.25**	.45***	(.84)			
14. Openness	.02	.02	.07	.17*	.07	− .12	− .07	.10	.11	− .23**	.14	.21*	.27**	(.63^b^)		
15. Agreeableness	.21**	.07	.17	.08	.10	.17	− .08	.10	.24*	− .10	.27*	.20*	.26**	.22*	(.80)	
16. Conscientiousness	.08	.24**	.00	.06	.04	.11	.09	.09	.05	− .08	.14	.25**	.19*	.09	.14	(.89)

Ordinal alphas are presented in parenthesis on the diagonal axis

***p < .001; **p < .01; *p < .05

^a^Average Polychoric R = .21

^b^Non-ordinal alpha = .72

Evidence for discriminant validity was also found as DS-R Animal-reminder Disgust, SQ-R15, and NEO-FFI Extraversion and Openness subscales did not significantly correlate with PVD factors. Likewise, NEO-FFI Agreeableness and Conscientiousness subscales showed low correlations with PI and GA, respectively.

### Reliability

Both factors showed good levels of internal consistency [41], Ordinal Cronbach's α_PI_ = 0.82, G6(smc)_PI_ = 0.81_,_ Median r_PI_ = 0.54, Ordinal Cronbach's α_GA_ = 0.82, G6(smc)_GA_ = 0.80, Median r_GA_ = 0.45.

## Discussion

The present study explored the psychometric properties of the PVD with a nonclinical sample of the Portuguese population. Results support the bifactorial structure proposed by several authors (e.g., [39, 42]), including Duncan and colleagues, which measures two conceptually distinct factors modestly intercorrelated: Perceived Infectability and Germ Aversion. Accordingly, PI seems to be more associated with rational appraisals, while GA reflects behavioral and emotional reactivity [8].

Although the original scale was comprised of 15 items, five problematic items were identified and, consequently, removed. Further analysis confirmed that these items did not reflect the latent factor they were supposed to be measuring. Regarding items Q5 and Q13, their reverse wording, which makes them harder to comprehend, might have been precluding participants from fully understanding their contents and causing the respective problematic loadings. In fact, both items have also been removed in other validation studies ([40], e.g., [43]). Furthermore, while most GA items mention behaviors or stimuli that, although from a broad spectrum, are commonly associated with disease (e.g., sharing a water bottle, washing/dirty hands), Q9’s content (i.e., wearing used clothes) appears to be the least directly disease-related item, which might explain the low loadings found. Likewise, despite sharing similar wording and content with the rest of the PI items, Q2 and Q6 still reached inadequate loadings. As the remaining items adequately measure the construct, both items were removed. Finally, another possible explanation is that the problematic loadings of all these items, from a conceptual point-of-view, may reflect cultural differences related to the prevalence of infectious diseases in different locations [8].

Thus, the aforementioned exclusion led to a stronger scale structure, when compared to various other models, with adequate goodness-of-fit indices, evidence for convergent and discriminant validity and good internal consistency levels for both factors. In particular, the GA factor obtained a reliability score higher than the one commonly found in the literature (e.g., [8, 43]).

Evidence for convergent validity of the scale was also found. First, both DPSS-R subscales positively correlated with PVD factors, with DPSS-R Disgust Sensitivity subscale correlating more strongly with PI, and DPSS-R Disgust Propensity subscale with GA. In fact, disgust propensity refers to how easily people respond with disgust and is more associated with avoidant action tendencies to repugnant materials, whereas disgust sensitivity is concerned with how unpleasant the experience of disgust really is and is linked with more general emotional sensitivity [44, 45]. Accordingly, if we look at the items from both PVD subscales, GA items involve actions (e.g., sharing a water bottle), while PI items include thoughts/beliefs about developing an infectious disease (e.g., higher susceptibility). Thus, these results add to the literature by suggesting that PI is more strongly related to disgust sensitivity and confirm the predicted link between GA and disgust propensity. Likewise, as a measure of disgust propensity, DS-R was expected to correlate with both PVD factors, albeit more strongly with GA. However, only a positive and significant correlation was found for the latter. This correlation was found for the total score, and both Core Disgust and Contamination-based Disgust subscales (both related to threat of infection) [46]. These results provide further evidence of the link between disgust propensity and GA and, more notably, highlight the conceptual distinction between both PVD factors.

A positive and significant correlation between both PVD factors and MMPI-Hs, MOCI and NEO-FFI Neuroticism was also found, as predicted, further corroborating the convergent validity of the scale. In particular, MMPI-Hs was found to correlate more strongly with PI, not surprisingly given that both constructs measure beliefs about disease. Conversely, GA correlated more strongly with both MOCI, as expected, and NEO-FFI Neuroticism. Interestingly, positive correlations between MOCI and both DS-R Core Disgust and Contamination-based Disgust subscales (i.e., disgust propensity measures correlated with GA) were also found. Taken together, these findings suggest that obsessive–compulsive symptoms may be tied to GA through disgust propensity, which is in line with a recent study that suggested that GA plays a mediator role between disgust propensity and contamination-based OCD [47]. Thus, our results corroborate the connection between these constructs even further. Finally, the positive correlation found between NEO-FFI Neuroticism and both PVD factors supports the findings of Duncan and collaborators [8], but contradicts those of Díaz et al. [40]. Since neuroticism is characterized by a tendency to experience negative emotions and psychological distress [21], the stronger correlation found for GA might be explained by this factor’s latent association with emotional reactivity.

Regarding the discriminant validity of the scale, DS-R Animal-reminder Disgust, SQ-R15, and NEO-FFI Extraversion and Openness subscales were not correlated with PVD factors, as predicted. Likewise, low correlations were found between PVD factors and NEO-FFI Agreeableness and Conscientiousness. Thus, these results provide further evidence of the PVD’s discriminant validity.

Despite the encouraging results, this study has some limitations, like the use of a nonclinical university sample and the high proportion of women, which limits the generalizability of the findings. Furthermore, cultural differences are influenced by the prevalence of infectious diseases and, thus, might be implicated in one’s perceived vulnerability to disease [8]. Consequently, our results might only be representative of regions with similar geographies or climates. Future research should use a more diverse and gender-balanced sample to further extend these results. Using a clinical sample would also be valuable to explore how one’s own health (physical or psychological) might influence perceived vulnerability to disease. Lastly, temporal stability analysis should also be explored.

## Conclusion

Individual differences in perceived vulnerability to infectious diseases are involved in disease avoidance responses and have implications for various psychological outcomes. This research aimed to analyze the psychometric properties of the PVD for the Portuguese population. Overall, the 10-items bifactorial solution of the European-Portuguese PVD appears to be a reliable and valid measure of one’s perceived vulnerability to disease. Thus, this instrument may offer a significant contribute to study social cognition and behavioral processes related to disease avoidance.

## Supplementary Information

**Additional file 1**: Full analysis outline and results.

**Additional file 2**: Original and Portuguese PVD items.

## Data Availability

The dataset and additional materials supporting the conclusions of this article are available in the Open Science Framework repository, through a view-only link at https://osf.io/hb48s/?view_only=c6bf2dfabd784a89977151c46581c62a.

## Abbreviations

AVE: Average Variance Extracted
BIS: Behavioral Immune System
CFA: Confirmatory Factor Analysis
CFI: Comparative Fit Index
CL: Cross-loading
CR: Composite Reliability
DPSS-R: Disgust Propensity and Sensitivity Scale-Revised
DS-R: Disgust Scale-Revised
EFA: Exploratory Factor Analysis
GA: Germ Aversion
KMO: Kaiser–Meyer–Olkin Measure of Sampling Adequacy
MMPI-2 Hs: Minnesota Multiphasic Personality Inventory–2 Hypochondria subscale
MOCI: Maudsley Obsessive Compulsive Inventory
NEO-FFI: NEO-Five Factor Inventory
PI: Perceived Infectability
PVD: Perceived Vulnerability to Disease Questionnaire
RMSEA: Root Mean Square Error of Approximation
SPQ-R15: Spider Phobia Questionnaire-Revised
SRMR: Standardized Root-Mean-Residual
TLI: Tucker-Lewis Index
ULS: Unweighted Least-squares Estimator
WLSMV: Weighted Least-square-mean and Variance Adjusted Estimator
χ^2^: Chi-square
