# Correction to: Perceived Vulnerability to Disease Questionnaire: Psychometric validation with a Portuguese sample

**DOI:** 10.1186/s40359-022-00859-9

**Published:** 2022-06-15

**Authors:** Jacqueline Ferreira, Ana C. Magalhães, Pedro Bem-Haja, Laura Alho, Carlos F. Silva, Sandra C. Soares

**Affiliations:** 1 CINTESIS@RISE, Department of Education and Psychology, University of Aveiro, Aveiro, Portugal; 2 William James Center for Research, University of Aveiro, Aveiro, Portugal; 3 Department of Education and Psychology, University of Aveiro, Campus Universitário de Santiago, 3810 193 Aveiro, Portugal; 4 Mind - Instituto de Psicologia Clínica e Forense, Lisbon, Portugal

## Correction to: BMC Psychology (2022) 10:1–10 https://doi.org/10.1186/s40359-022-00838-0

Following publication of the original article [1], it came to the authors' attention that the article had published with incorrect affiliations information in the author list: where any authors should have been assigned affiliation 3, they had been assigned affiliation 4; and where any authors had been assigned affiliation 4, they had been assigned affiliation 3. Moreover, in relation to this error, the authors also highlighted that the additional files of the article had published with erroneous affiliations information included.

The author list and the addition files have been corrected in the published article, and the corrected author list and additional files may be seen in this correction. Please note that the 'Author details' of the article is not affected by this correction.

## Supplementary Information

**Additional file 1** Full analysis outline and results.

**Additional file 2** Original and Portuguese PVD items.
