# Supplementary material for: Perceived Vulnerability to Disease Questionnaire: psychometric validation with a Portuguese sample
Source: BMC Psychol. 2022 May 22;10:130. doi: 10.1186/s40359-022-00838-0 (PMC9124547; doi:10.1186/s40359-022-00838-0)
Supplement: Supplementary file 2 — Additional file 2: Original and Portuguese PVD items. [file 40359_2022_838_MOESM2_ESM.docx]

**Perceived Vulnerability to Disease Questionnaire: Psychometric validation with a Portuguese sample**

Jacqueline Ferreira^1^, Ana C. Magalhães^2^, Pedro Bem-Haja^1^, Laura Alho^3^, Carlos F. Silva^2^, & Sandra C. Soares^2*^

^1^Center for Health Technology and Services Research (CINTESIS. UA), University of Aveiro, Aveiro, Portugal

^2^William James Center for Research, University of Aveiro, Aveiro, Portugal

^3^Mind - Instituto de Psicologia Clínica e Forense, Lisboa, Portugal

*Corresponding author: Sandra C. Soares, Department of Education and Psychology, University of Aveiro, Campus Universitário de Santiago, 3810-193, Aveiro, Portugal; Email: [sandra.soares@ua.pt](mailto:sandra.soares@ua.pt)

Additional File 1

| Table A  *Original and corresponding Portuguese items of the Perceived Vulnerability to Disease questionnaire, per factor* | | | |
| --- | --- | --- | --- |
| Factor | Item | Original version | Portuguese version |
| Perceived Infectability | Q2 | If an illness is ‘going around’, I will get it. | Se uma doença “anda por aí”, eu vou contraí-la.^1^ |
|  | Q5 | My past experiences make me believe I am not likely to get sick even when my friends are sick. (R) | As minhas experiências passadas fazem-me acreditar que não sou suscetível a adoecer mesmo quando os meus amigos estão doentes.^1^ (R) |
|  | Q6 | I have a history of susceptibility to infectious disease. | Tenho uma história de suscetibilidade às doenças infeciosas.^1^ |
|  | Q8 | In general, I am very susceptible to colds, flu and other infectious diseases. | No geral sou muito suscetível às constipações, às gripes e a outras doenças infeciosas. |
|  | Q10 | I am more likely than the people around me to catch an infectious disease. | Sou mais suscetível a contrair doenças infeciosas do que as pessoas à minha volta. |
|  | Q12 | I am unlikely to catch a cold, flu or other illness, even if it is ‘going around. (R) | Não sou suscetível a contrair constipações, gripes ou outras doenças infeciosas mesmo quando elas “andam por aí”. (R) |
|  | Q14 | My immune system protects me from most illnesses that other people get. (R) | O meu sistema imunitário protege-me da maioria das doenças que outras pessoas apanham. (R) |
| Germ Aversion | Q1 | It really bothers me when people sneeze without covering their mouths. | Incomoda-me muito quando as pessoas espirram sem tapar a boca. |
|  | Q3 | I am comfortable sharing a water bottle with a friend. (R) | Sinto-me confortável em partilhar uma garrafa de água com um amigo(a). (R) |
|  | Q4 | I do not like to write with a pencil someone else has obviously chewed on. | Não gosto de escrever com um lápis que foi visivelmente mordido por alguém. |
|  | Q7 | I prefer to wash my hands pretty soon after shaking someone’s hand. | Prefiro lavar as minhas mãos logo após cumprimentar alguém com um aperto de mão. |
|  | Q9 | I dislike wearing used clothes because you do not know what the last person who wore it was like. | Não gosto de usar roupas usadas porque não se sabe quem as usou.^1^ |
|  | Q11 | My hands do not feel dirty after touching money. (R) | Não sinto as minhas mãos sujas depois de tocar em dinheiro. (R) |
|  | Q13 | It does not make me anxious to be around sick people. (R) | Não me sinto ansioso (a) por estar próximo (a) de uma pessoa doente.^1^ (R) |
|  | Q15 | I avoid using public telephones because of the risk that I may catch something from the previous user. | Evito usar telefones públicos por causa do risco de contrair alguma coisa dos utilizadores anteriores. |

*Note*. ^1^Items excluded in the final Portuguese version of the scale; R = reverse items.
