# Supplementary material for: Correction to: Perceived Vulnerability to Disease Questionnaire: Psychometric validation with a Portuguese sample
Source: BMC Psychol. 2022 Jun 15;10:150. doi: 10.1186/s40359-022-00859-9 (PMC9199164; doi:10.1186/s40359-022-00859-9)
Supplement: Supplementary file 1 — Additional file 1 Full analysis outline and results. [file 40359_2022_859_MOESM1_ESM.pdf]

Additional File 1

Full Analysis Outline and Results

Analysis Outline

All analysis were performed using R (2020). The following packages were used: highr (Xie & Qiu, 2021), rio (Chan et al., 2021), psych (Revelle, 2021), GPArotation (Bernaards & Jennrich, 2005), EFAtools (Steiner & Grieder, 2020), readxl (Wickham & Bryan, 2019) and lavaan (Rosseel, 2012).

Mardia's Test was performed to assess Multivariate Normality of the sample (Mardia, 1970). The Jöreskog and Sörbom (1996, p. 171) equation (see below) was used to verify the adequacy of the sample size for the factor analysis.

$$Number\ of\ Participants = \frac{(p+1)(p+2)}{2}$$

where p is the number of observed variables.

1. Construct Validity

Construct validity of the Portuguese version of the PVD was evaluated by calculating its three sub-components: Factorial, convergent and discriminant validity.

1.1 Factorial Validity

There is great debate in the literature regarding the use of Exploratory Factor Analysis (EFA), Confirmatory Factor Analysis (CFA), or both, when carrying out structural validations (e.g., Bates et al., 2007). Since all items have been translated and adapted, and that versions from several countries show inconsistent factorial structures, an EFA followed by a CFA was conducted (Bagozzi et al., 1980). Several CFAs were then compared to verify the factor structure that best fits the data.

1.1.1 EFA

Considering the violation of normality assumption and the ordinal nature of the data, estimators based on the Asymptotic Covariance Matrix were used. These derived from

the Polychoric Correlation Matrix estimated from the observed categorical variables (Katsikatsou et al., 2012). To perform the EFA, the Unweighted Least-squares Estimator (ULS) estimator based on the diagonal form of the Asymptotic Covariance Matrix was used. This estimator has been shown to be more robust with smaller sample sizes (e.g., Forero et al., 2009). As a factor retention criterion, Parallel Analysis with ULS method, Kaiser criteria (1 or .7; old and new Kaiser Criterion, respectively), Hull method with Comparative Fit Index (CFI) and Root Mean Square Error of Approximation (RMSEA), and lower bound of RMSEA 90% CI were used. Since a correlation between the factors was expected, an oblique rotation approach was performed to allow for the factorial correlation. Promax rotation was chosen because it assumes the factors are orthogonal and then relaxes the rotation, allowing them to correlate (Russell, 2002). Considering our sample size (N = 136), loadings of .50 or greater were considered practically significant (see table 3.2, pp152, in Rajalahti & Kvalheim, 2011). Cross-loadings were considered when the difference between the primary and secondary factor loadings was below .3 (see Matsunaga, 2010) and when the ratio between these same loadings was below 2 (see Hair et al., 2009). Hofmann's (1978) complexity was also computed to discuss item cross-loadings. An EFA average with different oblique rotations models was performed to assess the fluctuation of item loadings and ensure elimination decision. The overall goodness-of-fit (and badness-of-fit) was assessed using the following indexes and cut-off points for "good adjustment": Chi-square ($\chi 2$); CFI ($.90 \leq CFI \leq .95$); Tucker-Lewis Index (TLI; $.90 \leq TLI \leq .95$); RMSEA ($.05 \leq RMSEA \leq .70$); P[rmsea $\leq$ 0.05]; and Standardized Root Mean Residual (SRMR; SRMR <.80) (see Marôco, 2014).

1.1.2 Polytomous Item Response Theory

Considering the elimination of items based on the EFA, we assessed whether these were important at discriminating the latent trait. Thus, two Polytomous Item Response

Theory analysis using Generalized Partial Credit Model - one for each factor (see model B of Toland et al., 2017) - were performed. Items with values above .7 were considered discriminatory (≥ .70; Embretson & Reise, 2000).

1.1.3 CFA

Given the violation of multivariate normality and the ordinal nature of the items, all CFA analysis were performed using Weighted Least Squares with Mean and Variance Adjustment (WLSMV; Finney & DiStefano, 2006). Global adjustment of the factor model was assessed using the same goodness-of-fit indices as in the EFA. Local adjustment was estimated by factor loadings/weights (high $\lambda \geq .5$) and individual reliability of the items (appropriate R2 ≥ .25). The various CFA models were compared using the adjustment indices and the item loadings (global and local adjustments of each model). In view of the non-normal distribution, comparative tests for non-nested models, such as those of Vuong (1989), were not possible.

1.2 Convergent validity

Convergent validity of each factor was assessed through the Fornell and Larcker (1981) method, and convergent validity of the measure with the classic correlational method (Campbell & Fiske, 1959).

1.2.1 Convergent validity of PVD factors

The Fornell and Larcker method involves calculating the Average Variance Extracted (AVE) and the Composite Reliability (CR) of each factor (see Hair et al., 2009). The AVE of each factor was calculated using the following formula:

$$\widehat{AVE_J} = \frac{\sum_{i=1}^{k} \lambda_{ij}^2}{\sum_{i=1}^{k} \lambda_{ij}^2 + \sum_{i=1}^{k} \varepsilon_{ij}}$$

(λ_{ij} are the standardized factor weights and $\varepsilon_{ij} = 1 - R_{ij}^2 \cong 1 - \lambda_{ij}^2$ are the residues of each item)

An AVE of .5 (which corresponds to an average loading of .7) indicates an adequate convergence between the items of each construct (Hair et al., 2009). Furthermore, CR was calculated using the following formula:

$$\widehat{CR}_J = \frac{\left(\sum_{i=1}^{k} \lambda_{ij}\right)^2}{\left(\sum_{i=1}^{k} \lambda_{ij}\right)^2 + \sum_{i=1}^{k} \varepsilon_{ij}}$$

A CR above .7 means that all items consistently represent the same latent construct (Hair et al., 2009).

1.2.2 Convergent validity of the scale

According to Campbell and Fiske (1959), a scale has convergent validity if it shows a significant correlation with a test that measures a trait theoretically related to what the scale measures. Thus, to show convergent validity[1], positive and significant Spearman correlations between PVD factors and both DPSS-R subscales, DS-R (total score, Core Disgust and Contamination-based Disgust subscales), MOCI, MMPI-2 Hs and NEO-FFI Neuroticism were expected. Particularly, a stronger correlation between GA and disgust propensity (i.e., DPSS-R Disgust Propensity, and DS-R subscales, especially Contamination-based Disgust), GA and MOCI, and PI and MMPI-Hs was expected.

1.3 Discriminant validity

Discriminant validity between the two factors was evaluated using the Fornell and Larcker method, and discriminant validity of the measure using the correlational method.

1.3.1 Discriminant validity of PVD factors

The discriminant validity between factors was calculated by comparing the AVE of each factor with the square of the correlation between the two factors (Anderson & Gerbing, 1988). If the individual AVE is greater than the square of the correlation between factors, discriminant validity between factors is obtained (Hair et al., 2009).

[1] See the theorical background for each hypothesis at the end of the document.

1.3.2 Discriminant validity of the scale

According to Campbell and Fiske (1959), a scale has discriminant validity if it shows a null correlation with a scale that measures a trait theoretically independent to what the scale measures. Thus, to show discriminant validity[2], low or non-significant Spearman correlations between PVD factors and DS-R Animal-reminder Disgust subscale, SPQ-R15 and NEO-FFI subscales (except Neuroticism) were expected.

2. Reliability

Reliability is the degree to which the items in the instrument covary, relative to their sum score (Boateng et al., 2018; Fenn et al., 2020). Thus, to test the reliability of the scale, and considering the ordinal nature of the data, internal consistency was assessed through calculation of Ordinal Cronbach's α (Zumbo et al., 2007), based on the Polychoric Matrix.

[2] See the theorical background for each hypothesis at the end of the document.

Results

1. Construct Validity

1.1 Factorial Validity

Results from the Mardia test showed that data is not multivariate normal, g1p = 34.23, $\chi_{Skew} = 1243.78$, $p < .001$; g2p = 281.09, $Z_{Kurtosis} = 8.53$, $p < .001$; $\chi_{SMSkew} = 1263.08$, $p < .001$. Sample size was considered adequate for factorial analysis.

Bartlett's test of sphericity was significant, $\chi^2(105) = 599.93$, $p < .001$, indicating that the matrix of population correlations is statistically different from the identity matrix. Furthermore, Kaiser-Meyer-Olkin Measure of Sampling Adequacy (KMO) analysis returned a value of .81 for the overall matrix, and values between .65 and .96 for all variables. Both indicators support factor analysis as a useful approach to the data.

Given non-normality and the ordinal nature of the data, Parallel Analysis with ULS estimator was conducted. Results suggest that three factors should be retained (see Figure 1). However, only two eigenvalues of principal factors registered values greater than 1 (old Kaiser Criterion) or .7 (new Keiser Criterion). Additionally, other retention criteria, such as the Hull Method with CFI and RMSEA, and the lower bound of RMSEA 90% CI, also support the retention of two factors. Given these results and the conceptual

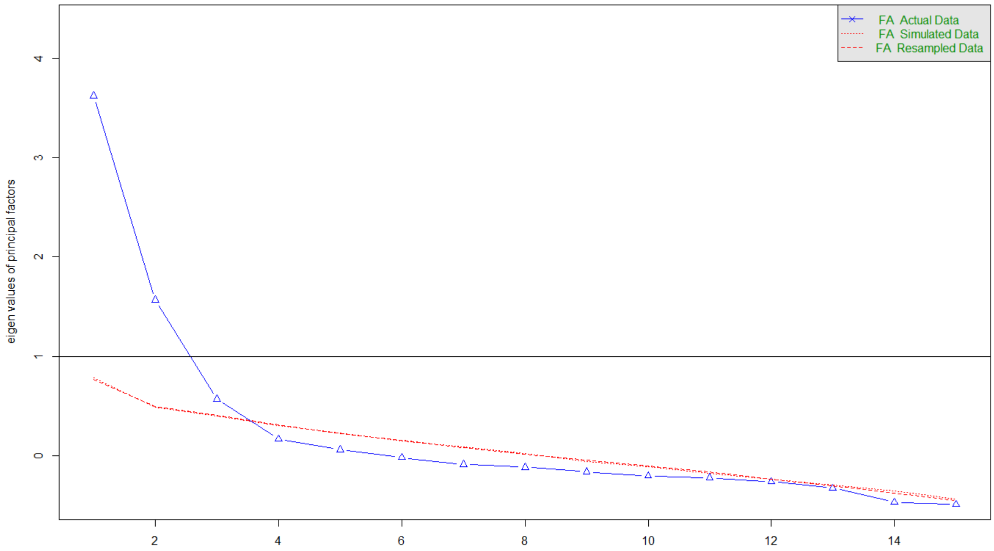

Figure 1. Parallel analysis scree plot with ULS estimator.

framework surrounding the two-factor structure of the original scale, a two-factor solution was extracted.

1.1.1 EFA

An EFA with Promax rotation and ULS estimator was performed. Factor loadings and respective R^2, Uniqueness and Complexity by factor are shown in Table 1, and goodness-of-fit indices are presented in Table 2.

Table 1

Factor loadings and respective R^2, uniqueness and complexity values.

	Factor					
	F1(PI)	F2(GA)	R^2	Uniqueness	Complexity	CL Ratio
Q1	-0.09	**0.68**	0.43	0.57	1.0	7.6
**Q2**	**0.43^LL^**	0.31	0.38	0.62	**1.8**	**1.4^CL^**
Q3	-0.15	**0.57**	0.29	0.71	1.1	3.8
Q4	0.01	**0.60**	0.37	0.63	1.0	60
**Q5**	**0.55**	-0.30	0.28	0.72	**1.6**	**1.8^CL^**
**Q6**	**0.44^LL^**	0.04	0.21	0.79	1.0	11
Q7	0.01	**0.68**	0.47	0.53	1.0	68
Q8	**0.84**	-0.13	0.64	0.36	1.1	6.5
**Q9**	0.01	**0.41^LL^**	0.17	0.83	1.0	41
Q10	**0.58**	0.22	0.47	0.53	1.3	2.6
Q11	0.00	**0.57**	0.33	0.67	1.0	57
Q12	**0.70**	0.01	0.49	0.51	1.0	70
**Q13**	0.19	**0.38^LL^**	0.23	0.77	**1.5**	**2^CL^**
Q14	**0.59**	0.07	0.38	0.62	1.0	8.4
Q15	0.05	**0.59**	0.37	0.63	1.0	11.8

Note: Items corresponding to each factor are listed according to the strength of their factor loading. PI=Perceived Infectability; GA=Germ Aversion; CL Ratio=Primary/Secondary Loading; LL=Loading below 0.5; CL=Cross-Loading.

The two extracted factors explained 37% of the variance, with the first factor (PI) accounting for 18% and the second factor (GA) for 19% of the variance. The inter-factor correlation was .35. Interestingly, this factorial solution mimics the two-factor solution expected and postulated in the literature, with the highest loading of each item saturated in the theoretically correct factor.

Items Q2 and Q13, in addition to having registered loadings below the recommended for the sample size (>.5), showed cross-loadings. In fact, both items registered discrepancies between the primary and secondary factor loadings above the recommended (difference < .3; see Matsunaga, 2010), a CL ratio above 2 (Hair et al., 2009) and complexity values above 1.5. Complexity above 1.5 means that, for each item, at least 1.5 latent variables are needed to adjust it (see Pettersson & Turkheimer, 2010). Despite an acceptable primary loading (> .5), item Q5 showed discrepancy between the primary and secondary factor loadings, as well as a CL ratio value that supports the existence of cross-loadings. In addition, 1.6 latent variables are needed to accommodate this item (see complexity value). Finally, items Q6 and Q9 have loadings below the recommended for the sample size. (> .5).

Table 2

Goodness-of-fit indices for the 15-item two-factor structure of the PVD.

	**RMSEA 90% CI**						
**RMSEA**	**Lower**	**Upper**	**RMSR**	**BIC**	**TLI**	**CFI**	**χ^2 df p**
.07	.04	.09	.06	-253.22	.88	.91	120.14 (76)<br>*p* = 0.00094

Regarding the global adjustment, goodness-of-fit indices showed an adequate adjustment for the two-factor model presented. The correlation between factors (r = .35) showed the need for an oblique rotation, thus validating the choice of Promax rotation (r > .32; Tabachnick & Fidell, 2001). Since the solution of the previous EFA requires the elimination of a large group of items (i.e., 5 items), an EFA AVERAGE combining the EFA results with the promax, oblimin and simplimax rotations was performed. The average, maximum and minimum results of the loadings in this analysis are shown in Figure 2.

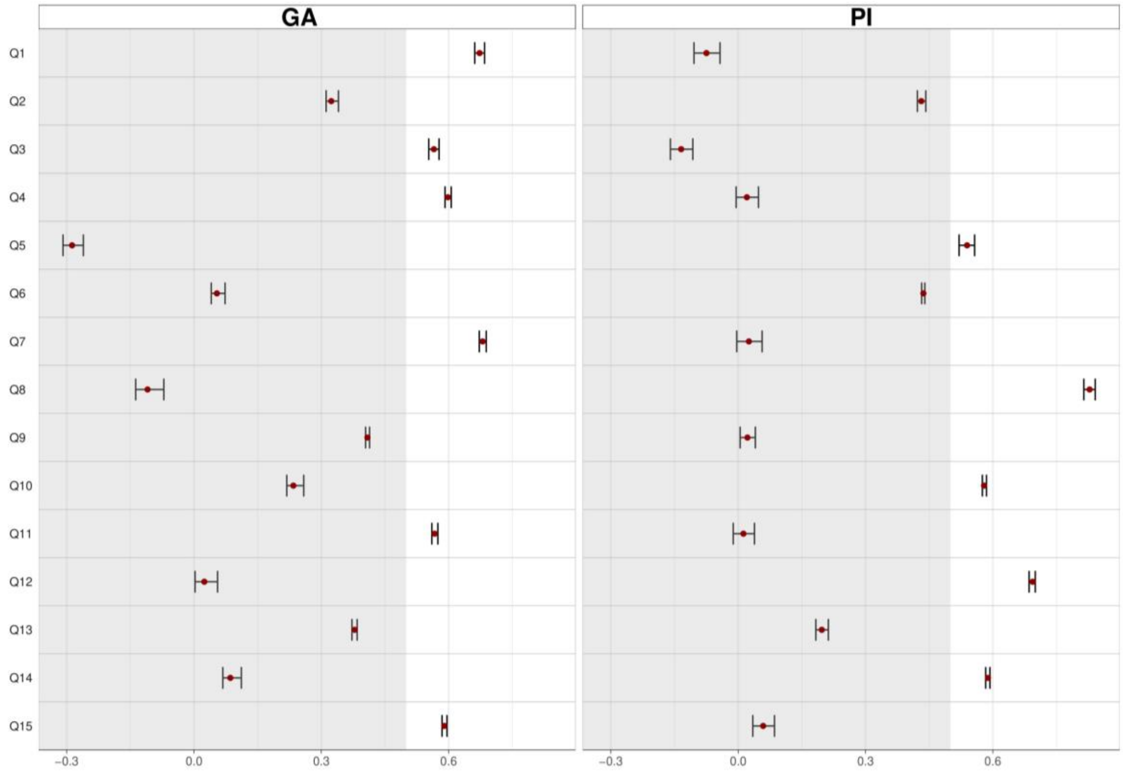

Figure 2. Average, maximum, and minimum loadings for each item per factor.

Note: GA = Germ Aversion; PI = Perceived Infectability.

Visual analysis of loadings distribution suggested an absence of major fluctuations, giving us more confidence in the elimination decision. Even for item Q5, while the average loading of the secondary factor is lower, the average loading of the primary factor is also lower, keeping the ratio between loadings (.539 – .287 = .252; difference < .0.3; see Matsunaga, 2010). Considering these results, the problematic items were eliminated.

1.1.2 PIRT

To evaluate if the deleted items had good discrimination ability of the latent trait, two PIRT analysis using Generalized Partial Credit Model - one for each factor - were performed. Results are shown in Table 3.

Table 3
Results of the PIRT analysis using Generalized Partial Credit Model per factor

	Item	a	b1	b2	b3	b4	b5	b6
PI	Q8	1.923	-1.291	0.412	0.555	0.648	1.405	2.168
	Q10	1.113	-0.902	1.497	0.326	2.984	0.719	2.37
	Q12	1.007	-3.341	-0.347	0.101	0.368	1.232	2.045
	Q14	0.7	-2.772	-0.5	-0.219	1.044	2.667	1.377
	**Q2**	0.562	-1.256	1.656	0.281	1.997	3.852	1.318
	**Q6**	0.425	-0.963	3.325	-0.416	2	6.297	-1.44
	**Q5**	0.31	-3.701	-0.189	-0.637	2.211	0.023	2.199
GA	Q1	1.234	-1.891	-1.952	-1.419	-1.734	-0.749	0.476
	Q7	1.093	-0.284	1.034	0.75	1.149	2.399	3.239
	Q15	0.513	0.298	0.961	-0.659	2.148	3.051	0.839
	Q4	0.465	-1.704	0.273	0.247	-0.689	0.237	1.611
	Q11	0.449	-1.279	0.417	1.284	-2	2.384	0.837
	Q3	0.422	-2.268	-0.802	0.348	1.044	0.262	0.067
	**Q13**	0.386	-3.475	0.881	1.476	0.234	1.961	4.371
	**Q9**	0.234	-1.348	0.674	0.614	0.395	0.104	1.327

Note: a = discrimination ability; PI=Perceived Infectability; GA=Germ Aversion.

In fact, data for parameter a (discrimination ability) showed that the items eliminated in the EFA (i.e., Q2, Q5, Q6, Q9, Q13) reached discrimination values below the acceptable (≥ 0.70; Embretson & Reise, 2000), indicating that they are not as good at discriminating the latent trait and giving us more confidence in their elimination.

1.1.3 CFA

To confirm the 10-items two-factor solution obtained in the EFA, a CFA with WLSMV estimator based on Polychoric Matrix was performed. This model (see Figure 3) revealed an acceptable global adjustment, $\chi2(34) = 46.681$, CFI = .934, TLI = .912, RMSEA = .053, RMSEA 90% CI [.00, .087], SRMR= 0.068. All the items reached high factor weights and appropriate individual reliabilities, suggesting a good local adjustment (Hair et al., 2009) and reflecting the latent factor being measured (Marôco, 2014).

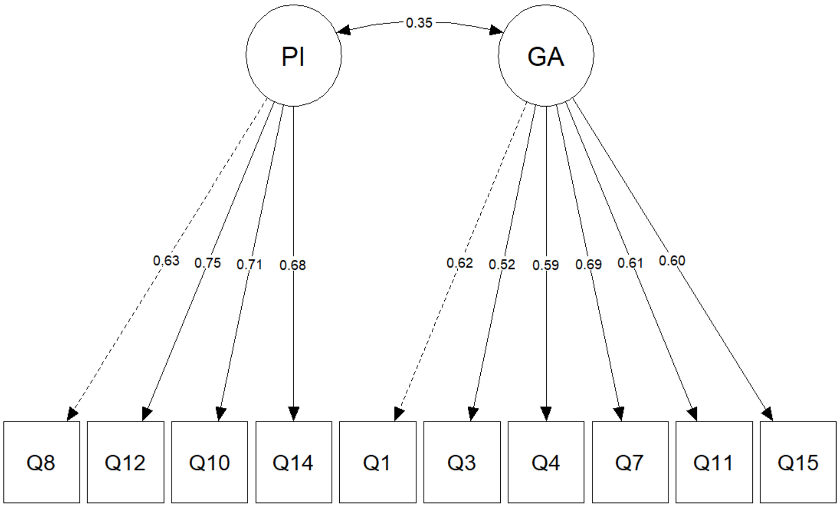

Figure 3. Diagram of two-factor structure (10 items) obtained using CFA with WLSMV estimator.
Note: PI = Perceived Infectability; GA = Germ Aversion.

A CFA was also conducted to assess the fit of the factor structure proposed by the original authors (i.e., without the removal of the five items). Furthermore, considering the cultural proximity, structure models from two studies that explored the psychometric properties of the PVD in the Spanish population were adopted and tested with our sample – Díaz and collaborators (2016) and Magallares and colleagues (2017). Since both studies use different estimators in their analysis (i.e., Díaz et al. uses the Maximum Likelihood method and Magallares et al., the WLS method), instead of the original estimators, we opted for the same one across all models, to allow for a more direct comparison. All models are present in Table 4.

Models 1, 2.1 and 3.1 obtained inadequate global and local adjustment values. Models 2.2 and 4.1, on the other hand, obtained acceptable values of global adjustment, but inappropriate local adjustment values. The remaining three models (i.e., 2.3, 3.2 and 4.2) reached acceptable values of global and local adjustments. Although these three models obtained adequate adjustment values, the bifactorial model resulting from the EFA (with the five items removed) was adopted because it has greater bonding with the conceptual framework and the structure of the original version of the questionnaire.

Table 4
Confirmatory Factor Analysis for all the models tested.

	Model 1	Model 2.1 Original	Model 2.2 Spain1	Model 2.3 Portugal	Model 3.1 Spain2	Model 3.2 Portugal	Model 4.1 Spain2	Model 4.2 Portugal
	One Factor PVD (all items)	Two Factors PI & GA (all items)	Two Factors PI & GA (w/o reverse items)	Two Factors PI & GA (w/o items Q2, Q5, Q6, Q9 and Q13)	One Factor PI (all items)	One Factor PI (w/o items Q2, Q5 and Q6)	One Factor GA (all items)	One Factor GA (w/o items Q9 and Q13)
χ2; p(df)	237.68; p<.001(90)	147.43; p<.001(89)	34.57; p=.12(26)	46.68; p=.07(34)	35.97; p=.001(14)	11.57; p<.01(2)	17.51; p=.62(20)	5.375; p=.80(9)
CFI	.50	.80	.95	.93	.84	.88	1.00	1.00
TLI	.42	.77	.93	.91	.76	.63	1.02	1.04
RMSEA (90%CI)	.11 (.09, .13)	.07 (.05, .09)	.05 (0, .09)	.05 (0, .09)	.11 (.07, .15)	.19 (.09, .30)	0 (0, .06)	0 (0, .06)
SRMR	.12	.09	.06	.07	.08	.07	.04	.03
Loadings Range	(.13 to .62)	PI (.28 to .75) GA (.42 to .70)	PI (.47 to .83) GA (.41 to .69)	PI (.63 to .75) GA (.52 to .70)	(.41 to .78)	(.61 to .79)	(.40 to .69)	(.55 to .67)
Items below 0.5	Q3, Q5, Q6, Q8, Q9, Q12, Q13, Q14	PI – Q5, Q6 GA – Q3, Q9	PI – Q6 GA – Q9		Q5		Q9, Q13	

Note: WLSMV was used as an estimator for all models. PI=Perceived Infectability; GA=Germ Aversion; Spain1= Study by Magallares and colleagues (2017); Spain2=Study by Díaz and collaborators (2016).

1.2 Convergent Validity

1.2.1 Convergent validity of PVD factors

The factors “Perceived Infectability” and “Germ Aversion” obtained AVE values of .414 and .334, respectively. These values are below those that are usually regarded as adequate (Hair et al., 1998). Regarding CR, the values obtained for the two factors were above .7, indicating an appropriate construct reliability ($CR_{perceived\ infectability}$ = .787 and $CR_{germ\ aversion}$ = .739). Despite the apparently inadequate AVE values, Fornell and Larcker (1981) state that if the AVE values are less than .5, but the CR values are higher than .6, convergent validity of the construct is still considered adequate. Thus, convergent validity of PVD factors was confirmed.

1.2.2 Convergent validity of the scale

Evidence for convergence validity of the scale was assessed through correlations with other validated measures. As shown in Table 5, both PVD subscales significantly correlated with DPSS-R subscales, MOCI, MMPI-Hs and NEO-FFI Neuroticism, while DS-R total score, Core Disgust and Contamination-based Disgust subscales only correlated with GA. Furthermore, DPSS-R Disgust Propensity subscale and MOCI correlated more strongly with GA, and MMPI-Hs with PI, as predicted.

1.3 Discriminant Validity

1.3.1 Discriminant validity of PVD factors

Results showed that AVE values of both factors were above the square of the correlation between the two factors (.123), which means that they only have 12.3% of common information between them and confirms the evidence of discriminant validity of the two-factor model (Fornell & Larcker, 1981; Malhotra, 2010).

1.3.2 Discriminant validity of the scale

Evidence for discriminant validity of the scale was also found (see Table 5) as DS-R Animal-reminder Disgust, SQ-R15, and NEO-FFI Extraversion and Openness subscales did not significantly correlate with PVD factors. Likewise, NEO-FFI Agreeableness and Conscientiousness subscales showed low correlations with PI and GA, respectively.

Table 5

Polychoric Correlation Matrix among study variables.

Variables	1	2	3	4	5	6	7	8	9	10	11	12	13	14	15	16
**PVD**																
1. Perceived Infectability	(.82)															
2. Germ Aversion	.27***	(.82)														
**DPSS-R**																
3. Disgust Propensity	.25**	.34***	(.85)													
4. Disgust Sensitivity	.29***	.22*	.50***	(.87)												
**DS-R**																
5. Core Disgust	.04	.28***	.31***	.40***	(.81)											
6. Animal-reminder	.02	.11	.20*	.39***	.68***	(.82)										
7. Contamination-based	.12	.41***	.16	.25**	.54***	.37***	(.58[1])									
8. Total	.00	.28***	.28***	.43***	.93***	.86***	.66***	(.89)								
**MOCI**																
9. Total	.39***	.42***	.43***	.35***	.18*	.12	.25**	.20*	(.86)							
**SQ-R15**																
10. Total	.12	.09	.38***	.27**	.30***	.18*	.15	.27**	.37***	(.89)						
**MMPI - Hs**																
11. Total	.32***	.23**	.41***	.27**	.13	.14	.10	.15	.52***	.29***	(.94)					
**NEO-FFI**																
12. Neuroticism	.26**	.17*	.32***	.35***	.20*	.12	.11	.18*	.54***	.37***	.57***	(.88)				
13. Extraversion	.15	.11	.10	.01	.06	.11	-.07	.06	.24**	-.08	.25**	.45***	(.84)			
14. Openness	.02	.02	.07	.17*	.07	-.12	-.07	.10	.11	-.23**	.14	.21*	.27**	(.63[2])		
15. Agreeableness	.21**	.07	.17	.08	.10	.17	-.08	.10	.24*	-.10	.27*	.20*	.26**	.22*	(.80)	
16. Conscientiousness	.08	.24**	.00	.06	.04	.11	.09	.09	.05	-.08	.14	.25**	.19*	.09	.14	(.89)

Note: Ordinal alphas are presented in parenthesis on the diagonal axis. ***p<.001; **p<.01; *p < .05; [1]Average Polychoric R=.21; [2]Non-ordinal alpha=.72.

2. Reliability

Results regarding internal consistency measures for each factor are presented in Table 6.

Both factors showed good internal consistency (Zumbo et al., 2007).

Table 6

Reliability Measures for both PVD factors

Factor 1: Perceived Infectability	
Ordinal Cronbach's α	.823
G6(smc)	.812
Median r (Polychoric correlation)	.544
Factor 2: Germ Aversion	
Ordinal Cronbach's α	.821
G6(smc)	.800
Median r (Polychoric correlation)	.451

Specific hypothesis for each validated measure used in the correlation analyses

Convergent validity

Given the close link between perceived vulnerability to disease and disgust, positive and significant correlations were expected between both PVD subscales, DPSS-R and DS-R. However, disgust is a construct comprised of different dimensions. For instance, disgust propensity refers to how easily people respond with disgust and is more associated with avoidant action tendencies to repugnant materials, while disgust sensitivity is concerned with how unpleasant the experience of disgust really is and is linked with more general emotional sensitivity (Fergus & Valentiner, 2009; Goetz et al., 2013; van Overveld et al., 2010). Although conceptually different, these two concepts are sometimes mistaken for one another. For instance, the DS-R was originally conceptualized as a measure of disgust sensitivity (Haidt et al., 1994; Olatunji, Williams, et al., 2007), but it appears to measure disgust propensity instead (Olatunji, Cisler, et al., 2007; van Overveld et al., 2006, 2008, 2010). Thus, while Duncan and collaborators (2009) mention a stronger correlation between disgust sensitivity and GA, they were actually measuring disgust propensity (and not disgust sensitivity) as they used the DS-R. With that in mind, we expected a stronger correlation between GA and disgust propensity in the form of DPSS-R Disgust Propensity, and DS-R subscales, especially Contamination-based Disgust (Díaz et al., 2016).

The PVD has been shown to correlate with fears related to death/disease (e.g., thanaphobia, hypochondria; Díaz et al., 2016; Duncan et al., 2009). Thus, a positive and significant correlation was expected between the MMPI-Hs subscale and both PVD subscales, particularly PI (Díaz et al., 2016).

Furthermore, despite the results of Díaz and collaborators (2016), a positive and significant correlation was also expected between MOCI and both PVD subscales,

particularly GA, given the established link between these constructs reported in the literature (Brady et al., 2021).

Finally, previous findings are not clear on the expected relationship between the Big Five personality traits and the PVD. While Duncan and colleagues (2009) found negative correlations between GA, Extraversion and Openness, and a positive correlation between Neuroticism, GA, and PI, Díaz and collaborators (2016) did not find any correlation between Extraversion or Neuroticism and both PVD factors. Since Neuroticism is closely linked to anxiety (Magalhães et al., 2014), which in turn has been shown to relate to pathogen threats (Fay et al., 2020), we expected a positive correlation between NEO-FFI Neuroticism, PI, and GA.

Discriminant validity

The DS-R Animal-reminder Disgust subscale is the only construct of this instrument not directly relevant to disease transmission (see Olatunji, Williams, et al., 2007). Thus, despite its link with disgust, low or non-significant correlations was expected between this subscale and PVD factors.

Likewise, since spider phobia is not related to disease, low or non-significant correlations were also expected between SQR-15 and PVD factors (Díaz et al., 2016).

Given the unclear relationships previously reported between personality traits and the PVD, low or non-significant correlations were expected between PVD factors and NEO-FFI Extraversion, Openness, Agreeableness and Conscientiousness.

References

Anderson, J. C., & Gerbing, D. W. (1988). Structural equation modeling in practice: A review and recommended two-step approach. *Psychological Bulletin*, *103*(3), 411–423. https://doi.org/10.1037/0033-2909.103.3.411

Bagozzi, R. P., Joreskog, K. G., Sorbom, D., & Magidson, J. (1980). Advances in factor analysis and structural equation models. *Journal of Marketing Research*, *17*(1), 133. https://doi.org/10.2307/3151129

Bates, R., Kauffeld, S., & Holton, E. F. (2007). Examining the factor structure and predictive ability of the German-version of the Learning Transfer Systems Inventory. *Journal of European Industrial Training*, *31*(3), 195–211. https://doi.org/10.1108/03090590710739278

Boateng, G. O., Neilands, T. B., Frongillo, E. A., Melgar-Quiñonez, H. R., & Young, S. L. (2018). Best Practices for Developing and Validating Scales for Health, Social, and Behavioral Research: A Primer. *Frontiers in Public Health*, *6*, 149. https://doi.org/10.3389/fpubh.2018.00149

Brady, R. E., Badour, C. L., Arega, E. A., Levy, J. J., & Adams, T. G. (2021). Evaluating the mediating effects of perceived vulnerability to disease in the relation between disgust and contamination-based OCD. *Journal of Anxiety Disorders*, *79*, 102384. https://doi.org/10.1016/j.janxdis.2021.102384

Campbell, D. T., & Fiske, D. W. (1959). Convergent and discriminant validation by the multitrait-multimethod matrix. *Psychological Bulletin*, *56*(2), 81–105. https://doi.org/10.1037/h0046016

Díaz, A., Soriano, J. F., & Beleña, Á. (2016). Perceived Vulnerability to Disease Questionnaire: Factor structure, psychometric properties and gender differences. *Personality and Individual Differences*, *101*, 42–49. https://doi.org/10.1016/j.paid.2016.05.036

Duncan, L. A., Schaller, M., & Park, J. H. (2009). Perceived vulnerability to disease: Development and validation of a 15-item self-report instrument. *Personality and Individual Differences*, *47*(6), 541–546. https://doi.org/10.1016/j.paid.2009.05.001

Embretson, S. E., & Reise, S. P. (2000). *Item response theory for psychologists*. Erlbaum Publishers.

Fay, A. J., Ainsworth, S. E., & Maner, J. K. (2020). State anxiety and pathogen cues jointly promote social cognitive responses to pathogen threats. *Social Cognition*,

38(1), 21–39. https://doi.org/10.1521/soco.2020.38.1.21

Fenn, J., Tan, C.-S., & George, S. (2020). Development, validation and translation of psychological tests. *BJPsych Advances*, *26*(5), 306–315. https://doi.org/10.1192/BJA.2020.33

Fergus, T. A., & Valentiner, D. P. (2009). The Disgust Propensity and Sensitivity Scale-Revised: An examination of a reduced-item version. *Journal of Anxiety Disorders*, *23*(5), 703–710. https://doi.org/10.1016/j.janxdis.2009.02.009

Finney, S. J., & DiStefano, C. (2006). Non-normal and categorical data in structural equation modeling. In G. R. Hancock & O. Mueller, Ralph (Eds.), *Structural Equation Modeling: A Second Course* (pp. 269–314). Information Age Publishing.

Forero, C. G., Maydeu-Olivares, A., & Gallardo-Pujol, D. (2009). Factor analysis with Ordinal indicators: A Monte Carlo Study comparing DWLS and ULS estimation. *Structural Equation Modeling: A Multidisciplinary Journal*, *16*(4), 625–641. https://doi.org/10.1080/10705510903203573

Fornell, C., & Larcker, D. F. (1981). Evaluating structural equation models with unobservable variables and measurement error. *Journal of Marketing Research*, *18*(1), 39–50. https://doi.org/10.1177/002224378101800104

Goetz, A. R., Lee, H. J., Cougle, J. R., & Turkel, J. E. (2013). Disgust propensity and sensitivity: Differential relationships with obsessive-compulsive symptoms and behavioral approach task performance. *Journal of Obsessive-Compulsive and Related Disorders*, *2*(4), 412–419. https://doi.org/10.1016/j.jocrd.2013.07.006

Haidt, J., McCauley, C., & Rozin, P. (1994). Individual differences in sensitivity to disgust: A scale sampling seven domains of disgust elicitors. *Personality and Individual Differences*, *16*(5), 701–713. https://doi.org/10.1016/0191-8869(94)90212-7

Hair, J. F., Anderson, R., Tatham, R., & Black, W. (1998). Multivariate data analysis. In *Technometrics* (5th ed.). Prentice Hall.

Hair, J. F., Black, W. C., Babin, B. J., & Anderson, R. E. (2009). *Multivariate data analysis: A global perspective* (7th ed.). Prentice Hall.

Hofmann, R. J. (1978). Complexity and simplicity as objective indices descriptive of factor solutions. *Multivariate Behavioral Research*, *13*(2), 247–250. https://doi.org/10.1207/s15327906mbr1302_9

Jöreskog, K. G., & Sörbom, D. (1996). *LISREL 8: User's reference guide*. Scientific Software International.

Katsikatsou, M., Moustaki, I., Yang-Wallentin, F., & Jöreskog, K. G. (2012). Pairwise likelihood estimation for factor analysis models with ordinal data. *Computational Statistics and Data Analysis*, *56*(12), 4243–4258. https://doi.org/10.1016/j.csda.2012.04.010

Magalhães, E., Salgueira, A., Gonzalez, A. J., Costa, J. J., Costa, M. J., Costa, P., & de Lima, M. P. (2014). NEO-FFI: Psychometric properties of a short personality inventory in Portuguese context. *Psicologia: Reflexao e Critica*, *27*(4), 642–657. https://doi.org/10.1590/1678-7153.201427405

Magallares, A., Fuster-Ruiz, M. J., & Morales, J. F. (2017). Psychometric properties and criterion validity of the Perceived Vulnerability to Disease Scale (PVD) in the Spanish population. *International Journal of Social Psychology*, *32*(1), 164–195. https://doi.org/10.1080/02134748.2016.1248025

Malhotra, N. (2010). *Marketing research: An applied orientation* (6th ed.). Bookman.

Mardia, K. V. (1970). Measures of multivariate skewness and kurtosis with applications. *Biometrika*, *57*(3), 519–530. https://doi.org/10.1093/biomet/57.3.519

Marôco, J. (2014). *Analysis of structural equations: Theoretical fundamentals, software & applications* (2nd ed.). REPORTNUMBER.

Matsunaga, M. (2010). How to factor-analysis your data: Do's, don'ts, and how-to's. *International Journal of Psychological Research*, *3*(1), 97–110. https://doi.org/10.21500/20112084.854

Olatunji, B. O., Cisler, J. M., Deacon, B. J., Connolly, K., & Lohr, J. M. (2007). The Disgust Propensity and Sensitivity Scale-Revised: Psychometric properties and specificity in relation to anxiety disorder symptoms. *Journal of Anxiety Disorders*, *21*(7), 918–930. https://doi.org/10.1016/j.janxdis.2006.12.005

Olatunji, B. O., Williams, N. L., Tolin, D. F., Abramowitz, J. S., Sawchuk, C. N., Lohr, J. M., & Elwood, L. S. (2007). The Disgust Scale: Item analysis, factor structure, and suggestions for refinement. *Psychological Assessment*, *19*(3), 281–297. https://doi.org/10.1037/1040-3590.19.3.281

Pettersson, E., & Turkheimer, E. (2010). Item selection, evaluation, and simple structure in personality data. *Journal of Research in Personality*, *44*(4), 407–420. https://doi.org/10.1016/j.jrp.2010.03.002

R Core Team. (2020). *R: A language and environment for statistical computing* (version 4.0.3). R Foundation for Statistical Computing.

Rajalahti, T., & Kvalheim, O. M. (2011). Multivariate data analysis in pharmaceutics: A

tutorial review. In *International Journal of Pharmaceutics* (7th ed., Vol. 417, Issues 1–2, pp. 280–290). Prentice Hall. https://doi.org/10.1016/j.ijpharm.2011.02.019

Russell, D. W. (2002). In search of underlying dimensions: The use (and abuse) of factor analysis in Personality and Social Psychology Bulletin. *Personality and Social Psychology Bulletin*, *28*(12), 1629–1646. https://doi.org/10.1177/014616702237645

Tabachnick, B. G., & Fidell, L. S. (2001). *Using multivariate statistics* (4th ed.). Allyn and Bacon.

Toland, M. D., Sulis, I., Giambona, F., Porcu, M., & Campbell, J. M. (2017). Introduction to bifactor polytomous item response theory analysis. *Journal of School Psychology*, *60*, 41–63. https://doi.org/10.1016/j.jsp.2016.11.001

van Overveld, M., de Jong, P. J., Peters, M. L., Cavanagh, K., & Davey, G. C. (2006). Disgust propensity and disgust sensitivity: Separate constructs that are differentially related to specific fears. *Personality and Individual Differences*, *41*(7), 1241–1252. https://doi.org/10.1016/j.paid.2006.04.021

van Overveld, M., de Jong, P. J., Peters, M. L., van Hout, W. J. P. J., & Bouman, T. K. (2008). An internet-based study on the relation between disgust sensitivity and emetophobia. *Journal of Anxiety Disorders*, *22*(3), 524–531. https://doi.org/10.1016/j.janxdis.2007.04.001

van Overveld, M., Jong, P. J. d., & Peters, M. L. (2010). The Disgust Propensity and Sensitivity Scale - Revised: Its predictive value for avoidance behavior. *Personality and Individual Differences*, *49*(7), 706–711. https://doi.org/10.1016/j.paid.2010.06.008

Vuong, Q. H. (1989). Likelihood Ratio Tests for Model Selection and Non-Nested Hypotheses. *Econometrica*, *57*(2), 307. https://doi.org/10.2307/1912557

Zumbo, B. D., Gadermann, A. M., & Zeisser, C. (2007). Ordinal versions of coefficients alpha and theta for likert rating scales. *Journal of Modern Applied Statistical Methods*, *6*(1), 21–29. https://doi.org/10.22237/jmasm/1177992180
