# Supplementary material for: Correction to: Perceived Vulnerability to Disease Questionnaire: Psychometric validation with a Portuguese sample
Source: BMC Psychol. 2022 Jun 15;10:150. doi: 10.1186/s40359-022-00859-9 (PMC9199164; doi:10.1186/s40359-022-00859-9)
Supplement: Supplementary file 2 — Additional file 2 Original and Portuguese PVD items. [file 40359_2022_859_MOESM2_ESM.pdf]

Additional File 2

Table A

Original and corresponding Portuguese items of the Perceived Vulnerability to Disease questionnaire, per factor

Factor	Item	Original version	Portuguese version
Perceived Infectability	Q2	If an illness is 'going around', I will get it.	Se uma doença "anda por aí", eu vou contraí-la.[1]
	Q5	My past experiences make me believe I am not likely to get sick even when my friends are sick. (R)	As minhas experiências passadas fazem-me acreditar que não sou suscetível a adoecer mesmo quando os meus amigos estão doentes.[1] (R)
	Q6	I have a history of susceptibility to infectious disease.	Tenho uma história de suscetibilidade às doenças infeciosas.[1]
	Q8	In general, I am very susceptible to colds, flu and other infectious diseases.	No geral sou muito suscetível às constipações, às gripes e a outras doenças infeciosas.
	Q10	I am more likely than the people around me to catch an infectious disease.	Sou mais suscetível a contrair doenças infeciosas do que as pessoas à minha volta.
	Q12	I am unlikely to catch a cold, flu or other illness, even if it is 'going around. (R)	Não sou suscetível a contrair constipações, gripes ou outras doenças infeciosas mesmo quando elas "andam por aí". (R)
	Q14	My immune system protects me from most illnesses that other people get. (R)	O meu sistema imunitário protege-me da maioria das doenças que outras pessoas apanham. (R)
Germ Aversion	Q1	It really bothers me when people sneeze without covering their mouths.	Incomoda-me muito quando as pessoas espirram sem tapar a boca.
	Q3	I am comfortable sharing a water bottle with a friend. (R)	Sinto-me confortável em partilhar uma garrafa de água com um amigo(a). (R)
	Q4	I do not like to write with a pencil someone else has obviously chewed on.	Não gosto de escrever com um lápis que foi visivelmente mordido por alguém.
	Q7	I prefer to wash my hands pretty soon after shaking someone's hand.	Prefiro lavar as minhas mãos logo após cumprimentar alguém com um aperto de mão.
	Q9	I dislike wearing used clothes because you do not know what the last person who wore it was like.	Não gosto de usar roupas usadas porque não se sabe quem as usou.[1]

	Q11	My hands do not feel dirty after touching money. (R)	Não sinto as minhas mãos sujas depois de tocar em dinheiro. (R)
	Q13	It does not make me anxious to be around sick people. (R)	Não me sinto ansioso (a) por estar próximo (a) de uma pessoa doente.[1] (R)
	Q15	I avoid using public telephones because of the risk that I may catch something from the previous user.	Evito usar telefones públicos por causa do risco de contrair alguma coisa dos utilizadores anteriores.

Note. [1]Items excluded in the final Portuguese version of the scale; R = reverse items.
